# Human Poisoning from Marine Toxins: Unknowns for Optimal Consumer Protection

**DOI:** 10.3390/toxins10080324

**Published:** 2018-08-09

**Authors:** Natalia Vilariño, M. Carmen Louzao, Paula Abal, Eva Cagide, Cristina Carrera, Mercedes R. Vieytes, Luis M. Botana

**Affiliations:** 1 Departamento de Farmacología, Facultad de Veterinaria, Universidade de Santiago de Compostela, 27002 Lugo, Spain; mcarmen.louzao@usc.es (M.C.L.); paula.abal@usc.es (P.A.); cristina.carrera@usc.es (C.C.); 2 Laboratorio CIFGA S.A., Plaza Santo Domingo 20-5°, 27001 Lugo, Spain; evacagide@cifga.es; 3 Hospital Veterinario Universitario Rof Codina, Facultad de Veterinaria, Universidade de Santiago de Compostela, 27002 Lugo, Spain; 4 Departamento de Fisiología, Facultad de Veterinaria, Universidade de Santiago de Compostela, 27002 Lugo, Spain; mmercedes.rodriguez@usc.es

**Keywords:** acute toxicity, chronic toxicity, toxicity equivalency factor, intoxication, biotoxin

## Abstract

Marine biotoxins are produced by aquatic microorganisms and accumulate in shellfish or finfish following the food web. These toxins usually reach human consumers by ingestion of contaminated seafood, although other exposure routes like inhalation or contact have also been reported and may cause serious illness. This review shows the current data regarding the symptoms of acute intoxication for several toxin classes, including paralytic toxins, amnesic toxins, ciguatoxins, brevetoxins, tetrodotoxins, diarrheic toxins, azaspiracids and palytoxins. The information available about chronic toxicity and relative potency of different analogs within a toxin class are also reported. The gaps of toxicological knowledge that should be studied to improve human health protection are discussed. In general, gathering of epidemiological data in humans, chronic toxicity studies and exploring relative potency by oral administration are critical to minimize human health risks related to these toxin classes in the near future.

## 1. Introduction

Marine toxins are globally distributed natural compounds produced by aquatic microorganisms that accumulate in shellfish or finfish through the food web and reach human consumers. There are several classes of marine biotoxins, such as saxitoxin (STX), domoic acid (DA), ciguatoxin (CTX), brevetoxin (BTX), tetrodotoxin (TTX), okadaic acid (OA), azaspiracid (AZA) and palytoxin (PLTX) groups. Many of them pose a serious threat to human health linked to poisoning after consumption of contaminated seafood, skin contact with contaminated water or inhalation of toxic aerosol. In spite of the high number of intoxications that occur every year and of the huge human and economic resources invested in protection plans, outreach to different sectors of society is needed to improve diagnosis and consumer awareness. We have reviewed the intoxication symptoms of the marine toxins with demonstrated human toxicity and discussed the gaps in toxicological information that should be explored to ensure adequate human health protection.

## 2. Saxitoxins

Saxitoxins, also known as paralytic shellfish toxins (PSTs), are neurotoxic alkaloids produced mainly by dinoflagellates of the genera *Alexandrium*, although production by the species *Pyrodinium bahamense* and *Gymnodinium catenatum* has also been reported [1]. PSTs cause human intoxications after ingestion of bivalve molluscs that feed by filtration on these phytoplanktonic producers [1,2]. Besides bivalve molluscs, other marine organisms have occasionally served as vectors of PSTs all the way through humans, including crustaceans, gastropods and some fish [1]. These toxins can be synthetized also by freshwater cyanobacteria [1,3,4]. Although sheep mortality has been described due to water consumption from a dam during a STX containing cyanobaceria bloom [5,6], no human intoxication has been reported from drinking water, probably due to the removal of 99.9% of the cyanobacterial cells by flocculation and sand filtration and, consequently, efficient reduction of PSP toxins during drinking water treatment [6].

**Figure 1:**
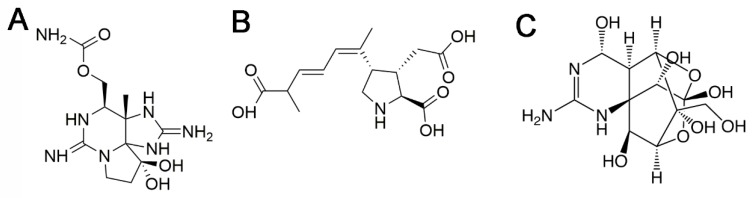
Chemical structure of saxitoxin (**A**), domoic acid (**B**) and tetrodotoxin (**C**).

This toxin class comprises at least 58 compounds [7]. The representative molecule of the group is saxitoxin (STX, Figure 1A). PSTs are classified attending to their chemical structure in several subgroups including carbamoyl derivatives (STX, neosaxitoxin (neoSTX), gonyautoxins1-4 (GTX1-4)), N-sulphocarbamoyl derivatives (GTX5, GTX6 and C1-4), decarbamoyl derivatives (decarbamoyl-STX (dc-STX), dc-neoSTX and dc-GTX1-4), and other less frequent deoxy-decarbamoylated, mono-hydroxy-benzoate, di-hydroxy benzoate and sulphated benzoate analogs, plus the freshwater *Lyngbya wollei* toxins and other PST analogs [7].

Saxitoxins have a worldwide distribution, with reports of PST-related toxicity in coastal regions of temperate and tropical areas [8,9].

PSTs block Na^+^ influx through the voltage-gated sodium channel (Na_v_) by binding to site 1 of the α subunit [10]. Therefore, these toxins inhibit the generation of action potentials in the membranes of neurons and muscle cells.

### 2.1. Acute Human Intoxication: Paralytic Shellfish Poisoning

Saxitoxins are responsible for the paralytic shellfish poisoning (PSP) syndrome. PSP symptoms start between 30 min and a few hours after ingestion of contaminated shellfish. Mild intoxication is manifested by a tingling sensation or numbness of the mouth (lips, tongue and gums) and face [9]. This paresthesia may progress to the neck, arms, fingertips, legs and toes. Headache, dizziness and nausea have also been reported in mild cases, although gastrointestinal symptoms are not common [9]. Severe PSP signs include ataxia, incoherence of speech, progression of a prickly sensation in the extremities to stiffness and incoordination, and a feeling of lightness. These symptoms may be accompanied by weakness, myalgia and respiratory difficulty. In very severe intoxications, muscular paralysis and labored breathing may evolve to respiratory arrest and death unless respiratory support is provided [3]. Death may occur as soon as 3–4 h after ingestion of contaminated seafood [3].

No antidotes are available [10]. Clinical management of PSP consists of palliative care until the toxin is eliminated, including fluid therapy and, in severe cases, respiratory support [3]. Individuals suffering severe poisoning that survive the first 24 h have a favorable prognosis and should make a full recovery in a few days.

Toxicokinetics information in humans has been gathered from PSP fatal victims. Post-mortem analysis of several tissues revealed a widespread distribution with PSTs being detected in the brain, cerebrospinal fluid, liver, bile, spleen, heart, kidneys, pancreas, lungs and thyroid and adrenal glands, which is also evidence of absorption in the gastrointestinal tract [11]. Although PSTs are capable of crossing the blood-brain barrier at high doses, it is not clear if they retain this ability at lower dosing levels [4]. In humans, there is also evidence of PST metabolism. The toxin profile found post-mortem in the gut of victims from PSP differ from the profile in urine, body fluids, spleen, pancreas, liver, kidney and lungs, suggesting the transformation of STX into neoSTX and dc-STX [11,12]. In vitro studies with human liver microsomes indicate that GTX2 and GTX3 may be metabolized by oxidation and glucuronidation to glucuronic-GTX2, glucuronic-GTX3, GTX1 and GTX4 [13].

A lowest-observed-adverse-effect level (LOAEL) for PSPs has been estimated around 1.5 µg of STX equivalents/kg b.w. [2]. However, there are some uncertainties in the original reports used for this estimation and some assumptions had to be made in order to be able to calculate a no-observed-adverse-effect level (NOAEL). Based on this estimate and a safety factor of 3 to calculate a NOAEL, an acute reference dose (ARfD) (the amount of a given toxin in food that can be ingested in a 24 h period without appreciable health risk to the consumer) of 0.5 µg of STX equivalents/kg b.w. was proposed [2]. Similarly, the joint FAO/IOC/WHO expert consultation report suggests an ARfD of 0.7 µg STX equivalents/kg b.w. [14]. Recently, the PST critical minimal dose has been re-estimated at 0.37 µg STX equivalents/kg b.w., with at least 10% of consumers exposed to this dose suffering PSP symptoms, regardless of the severity [15]. The authors discuss that an over-estimation of the risk is possible owed to reporting bias in detriment of non-symptomatic individuals, which are critical for a more reliable estimation of the risk. Mild symptoms would be expected in more than 10% of the population exposed to 1.85 µg STX equivalents/kg of body weight (b.w.). Severe poisoning has been reported at doses ranging from 5.6 to 2058 µg STX equivalents/kg [2]. Recent estimations suggest that the minimal dose for moderate and severe PSP poisoning in more than 10% of the individuals would be around 5.16 and 82.2 µg STX equivalents/kg b.w. respectively [15]. Amounts of 1–4 mg of STX are potentially lethal to humans [10]. The great variations in toxic and lethal doses are reported to depend mainly on differences of individual sensitivity [3]. Children are considered more sensitive to PST toxicity than adults [4].

### 2.2. Chronic Toxicity of Saxitoxins

Awareness of the possible continuous or repeated exposure of humans to low amounts of PSTs has raised concerns in the scientific community about the chronic effects that this exposure might have on human health [4]. Not many studies on chronic toxicity of these toxins are available, and no data has been reported in humans. Repeated exposure of rats to subacute doses of neoSTX for 12 weeks (6 µg/kg, once daily, subcutaneous) caused a reduction of body weight and food intake during the treatment period and the animals recovered quickly after the treatment was interrupted. No other behavioral changes were observed and no damage to several organs was detected by histological evaluation [16]. However, serum bilirubin, γ-glutamyltransferase (GGT) and aspartate aminotransferase (AST, formerly SGOT), three markers of liver damage, were elevated after 12 weeks of neoSTX treatment. These changes might reflect the detoxifying effort or be a consequence of the food intake reduction [16]. Lower doses (half and 1/6) had no effect at all.

In another study, rats exposed to cyanobacterial cultures at final concentrations of 3 and 9 µg/L of STX equivalents in drinking water, with estimated intakes of 0.24 and 0.72 µg/day respectively, did not show clinical signs of toxicity, but had altered antioxidant defenses and biochemical signs of oxidative stress in brain and liver [17]. In a later publication the same authors indicated that rats exposed to the 9 µg STX equivalents/L concentration had a statistically significant worse performance in behavioral memory tests than controls [18]. Although this second article includes a better description of the toxin content of the cyanobacterial culture than the first one, stating that the preparation contained STX, neoSTX and dc-STX, the exact amount of each analog present or how they estimated STX equivalents was not detailed. An alteration of antioxidant enzymes and xenobiotic-metabolizing enzymes was also observed in livers of mice that received three oral administrations every 3 days of GTX2/3 sublethal doses (300 and 200 µg/kg of GTX2 and GTX3 respectively) [19].

Overall, PSTs do not seem to have serious chronic toxic effects in mammals [16]. On the contrary, in humans it seems that previous exposure to these toxins lowers the sensitivity to the toxins in later intoxications [2,9].

### 2.3. Relative Toxic Potency of Saxitoxin Analogs

Relative toxicities of the different analogs of this toxin class in humans have not been obtained. This information is critical to evaluate toxicity of a sample with analytical methods [20,21]. Although the official reference method for detection of PSTs has been the mouse bioassay in the EU for a long time [22,23], there are several official analytical methods routinely used for their detection such as high-pressure liquid chromatography coupled to fluorescent detection (HPLC-FLD) [24,25]. Recently, a regulation has been published to replace the bioassay by the HPLC-FLD AOAC official method 2005.06 as reference method for PST detection, which shall apply from January 2019 [23]. This legislation change is clearly indicative of a trend to replace animal assays by analytical methods. Naturally contaminated shellfish usually contain a mixture of several PST analogs. Analytical methods allow the quantification of the analogs present in the sample, but do not provide information about their toxicity. Therefore, the toxic potency of each analog is required to make an adequate estimation of shellfish toxicity. Owed to the absence of relative toxic potency data in humans, intraperitoneal (i.p.) toxicity in mammals [2] and in vitro toxicity in rodent neurons [26] were used to propose provisional toxicity equivalency factor (TEF) values by the European Food Safety Authority (EFSA) CONTAM Panel of experts for the more frequent PST analogs, with STX as the reference compound [2] (Table 1). Considering that the route of exposure of human consumers to these toxins is ingestion, oral toxicity data of PSTs would be more relevant to derive TEF values. After the EFSA report, oral toxicity in mice has been published for most PST analogs that have a TEF value proposed by EFSA. In these studies, the toxins were administered by oral gavage or voluntary ingestion. LD_50_ and NOAEL values were obtained for both administration methods and used to re-calculate relative toxicity factors (Table 1). Because the consistency of the stomach content in mice and humans is very different, toxicity by voluntary ingestion has been considered in a FAO/WHO technical paper as more relevant for the purpose of deriving TEF values [27]. The most remarkable difference with former i.p.-based TEF values is the increase of neoSTX TEF (1.7 for gavage, 2.54 for feeding), which was previously considered equipotent with STX [28]. In addition, there is also a decrease of TEF values of decarbamoyl toxins (Table 1). Re-evaluation of TEFs should be based on these data of PST oral toxicity. TEF values proposed in the joint FAO/WHO technical paper are based on oral toxicity when available, otherwise i.p. toxicity was used [27] (Table 1).

### 2.4. Limitations and Implications of Current Toxicological Information on Saxitoxins

In order to protect human health, the maximum content of PST in shellfish meat has been regulated in many countries [30]. The lack of information about chronic toxicity does not allow an estimation of TDI (tolerable daily intake) and, therefore, it has prompted the establishment of regulated PST limits based on the ARfD. According to Codex Alimentarius provisions, most regulations establish a maximum permitted content of PSTs in shellfish of 0.8 mg STX (2HCl) equivalents (eq)/kg of flesh in edible parts [31,32].

Although the evidence gathered so far seems to indicate that PSTs do not have remarkable chronic toxicity, the studies currently available are very scarce, and all the data have been obtained from animals. Actually, previous exposure seems to have a protective effect against later intoxications. However, neither of these two characteristics of PST toxicity have been undoubtedly demonstrated in humans, and therefore the irrelevance of a TDI estimation has not been determined.

Implementation of these regulations to ensure consumer safety requires monitoring and detection of PSTs in shellfish. As mentioned above, TEF values for all analogs present in the sample are necessary for adequate estimation of sample toxicity by analytical methods. In spite of the recent oral toxicity results, information about oral toxicity of less frequent analogs is still lacking. Actually, oral LD_50_ values are available for 18 of the 58 analogs of the group, and for 10 of those 18 analogs, GTX1&4, GTX2&3, dc-GTX2&3, C1&2 and C3&4, only the oral toxicity of equilibrium mixtures has been reported. Therefore, it will be necessary in the near future to determine relative oral toxicity for most PST analogs in order to improve food safety. Monitoring programs have reduced considerably the number of marine toxin outbreaks, and seafood safety should be maintained or improved by adaptation of these programs to new regulations.

Toxicokinetics of PSTs demonstrate that these toxins are metabolized in humans, and in some cases the products of this metabolism may be more toxic than the parent toxin (transformation of STX to neoSTX, or GTX2&3 to GTX1&4, see Table 1 and [11,13,28]). However, for most PSTs, metabolism in humans is unknown. With regards to animal data, it is important to note that metabolic routes differ greatly among species and, therefore, using animal models in this instance has some limitations [4]. Collection of samples from PSP victims intoxicated by ingestion of shellfish with varying toxin profiles is critical to explore metabolism of other PSTs. 

## 3. Domoic Acid

Domoic acid (DA, Figure 1B) is a naturally occurring excitotoxin originally isolated from the marine red alga *Chondria armata* and produced by the diatoms *Nitzschia*, *Pseudo-nitzschia* and *Amphora* [33,34]. These toxic diatoms are found in polar, temperate, subtropical and tropical regions and are responsible for many toxic blooms worldwide affecting coastal environments in Europe, America or Oceania [35]. DA accumulates in filter-feeding shellfish that become harmful to wildlife and humans that consume them causing the amnesic shellfish poisoning (ASP). The first poisoning related to DA that caused the death of 153 people after consumption of contaminated mussels occurred in Prince Edward Island (Canada) in 1987 [36]. Vectors are clams, mussels, oysters, scallops but also squid, sardines, anchovies, crab and lobster [37]. Clams can hold the toxin for up to 1 year in the natural environment, or several years after being processed, canned or frozen [38].

DA has a low bioavailability and it is rapidly eliminated in the kidney of rats [39]. However, when DA reaches the brain, it damages the hippocampus and the amygdala by activating the alphaamino-5-methyl-3-hydroxyisoxazole-4-propionate (AMPA) and kainate receptors of neurons, decreasing the ATP levels and starting an uncontrolled influx of calcium that triggers cell degeneration [40,41]. DA also causes neurotoxicity through *N*-methyl-d-aspartate (NMDA) receptor activation [42].

### 3.1. Acute Human Intoxication: Amnesic Shellfish Poisoning

DA induces neurologic but also gastrointestinal symptoms in humans; however, the problem is limited compared to other marine toxins. Besides, some studies show that DA may have endocrine effects that relate to water balance and renal damage [27]. The evaluation of DA concentration in shellfish is performed in many countries regularly but the exposure assessment of consumers to DA is scarce and the human ASP reported cases are very rare [43]. Clinical diagnosis of ASP is largely based upon a detailed history of recent shellfish consumption and symptoms that begin from 15 min to 48 h after ingestion. Gastrointestinal symptoms including nausea, vomiting, diarrhea or abdominal cramps appear within 24 h after the ingestion of contaminated shellfish. Then, in more severe cases, neurological symptoms such as confusion, disorientation, headache, memory loss, seizures, coma as well as hemodynamic instability and cardiac arrhythmias have been detected within the following 24 h [44]. Most patients improve within 24 h to 12 weeks, but when consumers develop serious neurological damage, they can die [45]. Autopsies of deceased individuals have shown brain damage characterized by neuronal necrosis and astrocytosis particularly in the hippocampus, a cerebral region associated with memory and learning, which leads to the diagnostic label: amnesic shellfish poisoning (ASP) [44,46,47]. Anterograde memory disorder was described as a prominent feature and was considered a hallmark of DA intoxication [48]. Males appeared to be more susceptible and increased age was identified as a risk factor for both memory loss and severity of the illness. Some clinical and histopathology data provide evidence of long term sequels of acute intoxication of DA in humans that can persist for several months [40].

DA is very hydrophilic and its potential toxicity is mitigated by its toxicokinetics, as it is poorly absorbed by the gut, exhibits relatively poor blood brain barrier (BBB) permeability and has a short half-life in most tissue compartments [40]. It has been established that DA is cleared almost exclusively by the kidney [40,49]. So far, only the pass of toxin through the placenta and the excretion in milk after i.v. and i.p. administration were proved [50]. Alterations in its toxicokinetics such as poorly developed BBB, age or renal diseases are risk factors for DA toxicity [51].

Treatment of ASP is supportive; respiratory support and correction of hemodynamic instability and cardiac dysrhythmias may be necessary.

For assessment of health risk, the ARfD has been established as the toxin in food expressed on body weight basis that can be ingested over a short period of time, usually during one meal or one day, without appreciable health risk to the consumer on the basis of all known facts at the time of evaluation. The European Food Safety Authority (EFSA) CONTAM Panel used the LOAEL estimated as 0.9 mg DA/kg for neurotoxicity in humans and established an ARfD of 30 µg/kg b.w. [52]. By using different uncertainty factors, the FAO/IOC/WHO Panel proposed a provisional ARfD of 100 µg/kg b.w. It was argued that the cumulative effects of low doses of DA are unlikely and it was considered that this dose may also be a provisional TDI [53].

### 3.2. Chronic Toxicity of Domoic Acid

DA may be toxic in animals and humans through the long-term ingestion of subacute amounts [50,54]. Subchronic (64 days) oral administration by gavage of DA 5 mg/kg/day to adult rats did not induce clinical changes but revealed morphological changes in the hippocampus of the animals [40]. This dose was equivalent to the estimated maximum dose of human exposure during ASP incidents. Some reports provide additional evidence of the long-term harmful effects of DA exposure of sea lions in their natural habitat [55]. Clinical signs included seizures, marked lethargy, inappetence, vomiting, muscular twitching, central blindness and abnormal behavior. Histological examination of the chronic neurological cases that died showed chronic lesions in the hippocampus and parahippocampal gyrus. These chronic neuropathology lesions were interpreted as likely due to a combination of DA exposure and the effect of ongoing seizure activity. A key observation is that these chronic effects were more often seen in juvenile animals supporting the view of higher susceptibility of the developing brain to DA exposure [56].

In humans, Grattan et al., in 2016, found that coastal dwelling Native Americans who ate more than 15 razor clams per month with less than 20 mg/kg DA over 4 years suffered mild memory decline, a symptom of DA neurotoxicity [57,58]. Besides, some studies have found everyday memory to be dependent upon the integrity of the hippocampus and the temporal lobes, which are cerebral structures disrupted by acute DA exposure [59]. A recent human health study established that high razor clams (with safe levels of DA) consumers would have worse everyday memory than non-consumers or low consumers based upon dietary exposure 10 days and 1year prior to assessment [46]. Therefore, not only short term but also long-term DA neurotoxicity may be associated with low level, chronic exposures in adults who are heavy consumers of bivalves.

### 3.3. Relative Toxic Potency of Domoic Acid Analogs

HPLC with UV detection is the official method to monitor DA in shellfish in the European Union [22]. To estimate the total toxicity of seafood samples there is a need to know the relative toxicity of regulated DA analogues [60]. This requires the determination of toxicity equivalency factors (TEFs) defined as the ratio between the toxicity of each analogue and that of the reference compound within the same toxin group [20]. Several isomers of DA and isodomoic acids A, B, C, D, E, F, G and H have been reported but most of them have not been detected in shellfish tissue [52]. Isodomoic acids D, E and F are found in shellfish but they are currently considered non-toxic [27]. Taking into account that the isodomoic acids occur in shellfish at much lower concentrations and are considered to be less toxic than DA, the CONTAM Panel concluded that setting of TEFs was not required for these toxins [52]. DA transforms to epi-DA and in general both have been shown to be of equal toxicological relevance. Hence, TEF = 1 is applicable to the sum of DA and epi-DA expressed as DA [20].

### 3.4. Limitations and Implications of Current Toxicological Information on Domoic Acid

The CODEX Committee on Fish and Fishery Products (CCFFP) has developed the Standard for Live and Raw Bivalve Molluscs and identifies the maximum level in mollusc flesh for DA as 20 mg/kg shellfish meat [32]; taking into consideration that consumption of 400 g of shellfish meat would result in an exposure of 8 mg DA (equivalent to about 100 µg DA/kg b.w. for a 80 kg adult, similar to the provisional ARfD proposed by FAO/IOC/WHO Panel).

The extent to which chronic low-level exposure impacts humans will be relevant to health risk assessment of DA and isomers in food. Neurotoxicological effects of chronic and subchronic oral exposure to low doses of DA should be better studied, particularly in pregnant woman, prenatal, very young individuals and elderly people.

Further investigations are required to assess the effect of DA in other tissues such as heart, the immune system, neuroendocrine system and the gastrointestinal tract.

Prevention of ASP includes coastal monitoring of water and shellfish to ensure consumer protection. When the level of DA is high there is a potential risk to consumers of aquaculture and fishery products, and therefore, beaches should be closed. Public health intervention, including case identification or surveillance, as well as health promotion to protect at risk communities is also a challenging albeit a necessary activity.

## 4. Ciguatoxins

Ciguatoxins are large cyclic polyether compounds produced by epibenthic dinoflagellates of the genera *Gambierdiscus* and *Fukuyoa* [61,62,63]. These organisms grow attached to macroalgae and coral surfaces in shallow waters and, therefore, ciguatoxins accumulate in herbivorous fish and, subsequently, in carnivorous fish following the food web. Ciguatoxins reach humans mainly through consumption of finfish, although they have also been reported in molluscs [64,65,66].

**Figure 2:**
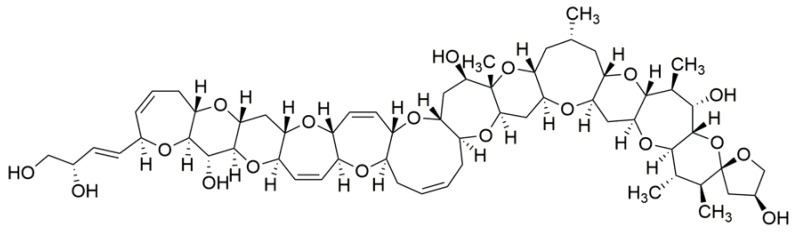
Chemical structure of Pacific ciguatoxin-1 (P-CTX-1).

These toxins have been classified into three groups that differ slightly in chemical structure: Pacific, Caribbean and Indian ciguatoxins [62]. Currently, more than 29 different analogs have been identified [62,67]. An annotation system has been adopted starting with P, C or I, depending on the origin of the toxin, followed by CTX from ciguatoxin, and ending with a number that indicates chronological order of description (see Table 2 for examples, Figure 2).

Human intoxication due to ingestion of ciguateric fish is the most frequently reported seafood-related poisoning [68]. Ciguatoxins are usually associated to tropical and subtropical waters of the Caribbean Sea, Pacific Islands and Indian Ocean [66,69,70,71]. However, there are several recent reports of ciguatoxin presence in fish from Canary Islands, Madeira and the temperate waters of Japan, South Korea or Israel, where they had not been described before [72,73,74,75,76,77]. Actually, international fish trade, tourism and seawater warming have important roles in the globalization of the risk that these toxins pose to human health [62,68,78].

Ciguatoxins activate voltage-dependent Na^+^ channels (Na_v_) by binding to site 5 in the α subunit and causing the opening of the ion pore at resting membrane potentials [79,80]. Na^+^ influx causes plasma membrane depolarization and triggers spontaneous action potentials.

### 4.1. Acute Human Intoxication: Ciguatera

Acute human intoxication with ciguatoxins is known as ciguatera or ciguatera fish poisoning (CFP). Clinical manifestations of ciguatera may vary depending on the geographical origin of the toxic fish, on the fish species (herbivore vs. carnivore), on the time from ingestion and on individual sensitivity, and include gastrointestinal, neurological and cardiovascular symptoms [68,80,81,82,83].

Signs of gastrointestinal (GI) toxicity appear approximately 0.5–12 h after ingestion of ciguateric fish, and they are more frequent in the acute phase of the Caribbean ciguatera [62,80]. Vomiting, nausea, abdominal pain and/or diarrhea have been reported. GI symptoms resolve in 1–4 days [68,69].

Neurologic symptoms are predominant in the acute phase of the Pacific and Indian Ocean ciguatera, although they commonly appear also in the Caribbean form of the disease [62,80]. Neurologic signs are usually present in the first 48 h after ingestion. One of the more frequent and characteristic neurological symptoms is cold allodynia, a misperception in which contact with cold surfaces triggers a burning or painful sensation. Other neurological signs associated with ciguatera include hand, feet or perioral paresthesias, dizziness, pruritus, taste alterations, arthralgia, myalgia, hypothermia, and headache [62,80,84]. Dental pain or loose teeth sensation have also been reported. Indian Ocean ciguatera is considered by some authors as a third form of ciguatera, with characteristic symptoms of incoordination, depression, hallucinations and nightmares being manifested in 16% of patients, in addition to the signs reported above [70,80]. In severe ciguatera cases, hallucinations, paralysis, ataxia, confusion and coma have been described [69,70,85,86]. Ciguateric fish are rarely deadly [68].

Alterations of the cardiovascular function consistent of hypotension and bradycardia may also be present concurrently with gastrointestinal and neurologic signs during the acute phase. Although cardiovascular symptoms are considered indicative of severe intoxication [87,88], recent retrospective studies suggest that their incidence is probably higher than initially reported [84].

An important feature of ciguatera is the permanence of neuropsychological symptoms for days or weeks, or even months and, in rare occasions, years, after the acute phase of the intoxication in some patients [68]. The prevalence of these long-term signs has been estimated at 20% [89]. They may include peripheral symptoms as cold allodynia, itching or paresthesias in the extremities; and/or central signs such as headache, memory loss, depression, anxiety, confusion, hypersomnolence or fatigue, among others [62,68,90]. The mechanism of long-term permanence of ciguatera symptoms is unknown and several hypotheses have been proposed, among them, the accumulation in adipose tissue of lipophilic ciguatoxins, the involvement of a ciguatoxin-triggered inflammatory response or slow elimination/long persistence of ciguatoxin in peripheral nerves [91].

Re-occurrence of neurological symptoms has been reported after ingestion of alcohol, non-ciguateric fish, chicken, pork or nuts, and also after exercise [68,80,82]. Interestingly, individuals that have suffered CFP seem to be sensitized to the toxin during posterior exposures, meaning the appearance of symptoms after ingestion of potentially ciguateric fish non-toxic for other people that ate from the same meal or more severe illness than in patients intoxicated for the first time [69,80,83,92]. However, sensitization is difficult to evaluate considering that the patient perception of the symptoms may be altered by previous poisoning and the re-occurrence of symptoms has also been reported after consumption of non-ciguateric or fresh water fish.

There is not much information about toxicokinetics of ciguatoxins in humans. Absorption in the gastrointestinal tract has been considered rapid and efficient, although diarrhea and vomiting probably contribute to toxin elimination before absorption. This assumption was made on the basis of the lipophilic nature of ciguatoxins, the rapid onset of neurological symptoms and the similarity of oral and i.p. toxicity in mice [65]. However, recent toxicokinetic studies in rats indicate that oral and i.p. bioavailabilities are not so similar, with values of 39 and 75% respectively [93]. These studies in rats confirm rapid absorption. CTXs also seem to be absorbed through the skin and mucous membranes, because there are reports of a tingling sensation in the hands after cleaning ciguateric fish or local pain after intercourse in sexual partners of ciguatera patients [65]. CTXs are excreted in breast milk, and probably in other secretions considering the apparent sexual transmission. CTXs can also cross the placenta [65]. In rats, CTXs distribute to several tissues, being detected in blood, liver, muscle and brain after oral administration [93]. In these studies, elimination occurred mainly in the feces, although P-CTX-1 was also detected in urine. Half-life of P-CTX-1 in rats is around 35.5 h after intravenous (i.v.) and 82 h after oral administrations [93,94]. No data about CTXs elimination is available in humans.

Human-to-human transmission of ciguatoxins has been described via breast feeding, trans-placental or sexual intercourse [67,68,83].

There is no specific treatment for ciguatera because no antidotes are available. CFP treatment is symptomatic and supportive [68,80]. Intravenous infusion of mannitol, which is considered to be more effective in the first 48–72 h, has been used as primary treatment to reverse, mainly, the neurologic symptoms [68,90]. However, i.v. manitol effectiveness in the treatment of ciguatera is controverted [95]. Supportive therapies are aimed at controlling dehydration and electrolyte imbalance (isotonic fluids), bradycardia (atropine), and respiratory failure (mechanical ventilation) [68,90,92]. Activated charcoal for toxin elimination and non-steroidal anti-inflammatory drugs for pain control have also been tried [68]. Vomiting and diarrhea contribute to toxin elimination and, therefore, are not usually suppressed, unless excessive or prolonged [68]. Although some drugs, such as pregabalin, amitriptyline, gabapentin or fluoxetine, seem to alleviate long-term symptoms after the acute phase [95], treatment of these symptoms is not recommended by some authors [68]. Actually, long treatments with these drugs are required to avoid symptom recurrence and there is not enough evidence at the moment to support their usefulness [95].

Pacific ciguatoxins are toxic at concentrations of 0.1 µg P-CTX-1 equivalents/kg in fish meat or higher [65,67,90]. From toxicological data the estimated dose that triggers mild ciguatera symptoms is 1 ng P-CTX-1/kg b.w. [65]. A lethal dose to humans has been estimated at 50 ng P-CTX-1/kg b.w. [67,96]. Caribbean ciguatoxins are considered less toxic than the Pacific analogs [62,65]. Concentrations of C-CTX-1 above 1 µg/kg of fish meat were estimated as the threshold for Caribbean ciguatera [76,97]. More recently, acute ciguatera has been reported for levels of C-CTX-1 equivalents in fish of 0.6 µg/kg [98]. Based on potency differences with P-CTX-1, for Caribbean ciguatoxin a dose of 10 ng C-CTX-1 equivalent/kg b.w. would be considered toxic in most individuals [67].

### 4.2. Ciguatoxin Chronic Toxicity

As mentioned above, it is well documented that neuropsychological signs may persist for long periods of time after acute exposure to ciguatoxins; however, there is no information about the effect of repeated exposure to subacute doses of these toxins. One of the hypothesis about sensitization and re-occurrence of neurological symptoms after apparent recovery is storage or sequestration of these lipophilic toxins in adipose tissue [65,80]. Considering this hypothesis, ciguatoxin accumulation in adipose tissue after repeated/continuous exposure to low doses would also be possible. No toxicokinetics studies have addressed this issue [65,67]. In addition, ciguatoxin effect in ex vivo tissue preparations remains long after toxin washout, suggesting “quasi-irreversible” binding to the sodium channel [91,99]. The possibility of chronic exposure to ciguatoxins having a deleterious effect on human health should not be dismissed.

The limited amount of pure ciguatoxins has precluded studies of repeated administrations for long periods of time. There is only one study of daily administration of P-CTX-1 and P-CTX-4C by oral gavage and by i.p. injection for 15 days. In these experiments, which were focused on heart toxicity, a dose of 0.1 µg/kg did not cause morphological alterations in the heart after a single administration, but after 15 doses, cardiomyocyte and endothelium damage were reported. Signs of heart tissue remodeling with deposition of collagen in interstitial spaces and bilateral ventricular hypertrophy remained 14 months later [100]. Heart alterations were similar to those observed after a single dose of 0.7 µg/kg. Unfortunately, the authors did not report if the mice had any toxicity symptoms or if any deaths occurred; although, the statement in the Methods section: “after these treatments all surviving mice…”, suggests that these treatments were lethal for some individuals [100]. In another study, i.p. administration of 0.26 µg/kg, which was reported as the “maximum tolerable dose”, induced a clear decrease of motor activity and temperature, and two administrations of the same dose caused a 30% mortality [101]. Therefore, it is not clear if the 0.1 µg/kg P-CTX-1 dose used in the first study could be considered a subacute dose, and thus, indicative of the risks related to low levels of toxin exposure in humans. In any case, this study highlights the possibility of accumulative effects of ciguatoxins after repeated exposure.

### 4.3. Relative Toxic Potency of Ciguatoxin Analogs

Although P-CTX-1 seems to be the predominant ciguatoxin in Pacific ciguateric fish, and the main analog responsible for ciguatera symptoms in the Pacific form of this illness [62,65]; in the toxin profile of Caribbean ciguateric fish there is no dominance of a single analog [62], and human intoxication cannot be attributed mainly to a single ciguatoxin. Therefore, the relative toxicity of ciguatoxin analogs to humans is unknown and could not possibly be derived at the moment from human intoxication data. As for other toxin groups, this information would be critical in order to estimate fish toxicity using analytical methods with the purpose of human health protection. This limitation has prompted the use of toxicological data obtained in animals to study relative potencies of different ciguatoxins. Oral toxicity would be more adequate because human exposure to these toxins occurs mainly through ingestion [65]; however, oral toxicity data are available only for P-CTX-1 and P-CTX-4C [67,100,102]. In these studies, both toxins seem to be equipotent. Nevertheless, the comparison seems to be based on symptomatology because a value of LD_50_ was not reported. In addition, criteria for symptom occurrence and intensity scoring were not presented. The same discussion should be applied to the comparison of i.p. and oral potencies of P-CTX-1 and P-CTX-4C, which were also reported as similar.

Intraperitoneal toxicity studies in mice remain as the only alternative at the moment to make an estimation of relative toxic potency of ciguatoxins. The TEF values adopted by the CONTAM Panel of EFSA based on i.p. LD_50_ of several analogs are shown in Table 2 [67].

### 4.4. Limitations and Implications of Current Ciguatoxin Toxicity Data

Ciguatera is the most frequent seafood intoxication [68]. In addition to this high frequency, the relevance and duration of clinical symptoms and medical care required by ciguatera victims in many cases, shows a need for official control of ciguateric fish, mainly in endemic areas. Because efficient, reliable detection of ciguatoxins in fish routinely performed before consumption is still not possible, prevention and management programs are based on the avoidance of ciguateric fish (certain species) or fish capture in known ciguatoxic areas in most countries. Actually, capture of high-risk ciguateric fish species is banned in some endemic countries [68]. Moreover, an effort to improve reporting of CFP, in order to gather more epidemiological data and evaluate the real incidence of ciguatera, has also been supported by public health authorities of several countries with CFP being declared a “notifiable” disease [68]. Epidemiological information is important to obtain adequate estimates of ARfD, LOAEL and NOAEL values and to improve risk assessment. Public outreach of the risk associated with CTXs is essential to prevent ciguatera because many intoxication cases originate from fish captures during recreational or self-subsistence activities [68]. Although important in all marine toxin poisonings, in the case of ciguatoxins, due to the lack of efficient pre-consumption toxin detection and controls, awareness and education of healthcare providers is an especially important part of ciguatera management programs.

Currently, the maximum limit of CTXs in fish has been regulated in some countries. In Japan and Mexico, for example, the maximum content in fish must not exceed 0.025 MU (mouse units)/g, and in the USA, 10 ng of P-CTC-1 eq/kg and 100 ng of C-CTX-1 eq/kg for Pacific and Caribbean CTXs respectively [109,110,111]. However, the lack of certified standards for ciguatoxins is an important obstacle for implementation of legal regulations.

In addition, for many compounds of this toxin class there is no information about oral toxicity. For 10 CTXs, EFSA CONTAM Panel has proposed TEFs based on i.p. LD_50_ (Table 2); however, considering the differences in polarity of ciguatoxins [65], it is possible that i.p. TEFs differ from oral relative potencies for many of these compounds, owed mainly to toxicokinetic differences. Oral toxicity of CTXs should be explored, although the scarcity of pure CTXs and the unavailability of certified standard materials make these studies a difficult endeavor at the moment.

## 5. Brevetoxins

Brevetoxins (BTXs or PbTxs) are a large family of lipophilic heat-stable cyclic polyether compounds initially produced by the dinoflagellate *Karenia brevis.* These toxins are rapidly metabolized in many animals including shellfish, so a variety of different toxin profiles are responsible for toxicity in animals [112]. Also, BTXs can accumulate along the marine food web and cause massive fish, birds and marine mammal mortality in impacted areas [113]. In humans, the ingestion of shellfish that are contaminated with brevetoxins can trigger neurotoxic shellfish poisoning (NSP). Documented outbreaks of NSP have occurred in New Zealand, Australia and Japan and periodically in the Gulf of Mexico and along the east coast of the USA [112]. NSP has not been recorded in European waters, however, some species of the genus *Karenia* are common in UK and Ireland waters. Vectors are mussels, clams, whelks, conch, coquinas, oysters, scallops but also muscle, liver and stomach contents of some planktivorous fish. Shellfish themselves do not appear to be affected by the accumulation of BTXs [114]. Depuration time of BTXs in shellfish varies, but it is typically within 2 to 8 weeks. Oysters exhibit rapid accumulation and reduction of toxins, where other species such as clams show longer depuration times [115]. BTXs may persist in finfish, particularly livers, for more than a year after bloom cessation [114]. There is scarce information regarding the effects of product processing on the levels of brevetoxins in shellfish [113]. However, like many marine toxins, BTXs are not diminished by rinsing or cleaning and the toxins cannot be detected by taste, smell or changes in morphology of seafood. This puts consumers of non-commercial seafood at an increased risk since in many cases NSP is associated with recreationally-harvested shellfish collected during or post “red tide” blooms [116].

BTX-induced effects are mainly due to high affinity binding to receptor site 5 on the voltage-gated sodium channels inducing a prolongation of open time and an increase in sodium currents. This BTX action lowers the membrane depolarization threshold and causes neurons to fire repetitively [117]. Both nerve and muscle are depolarized even though in some cases only the nerve is depolarized [114].

### 5.1. Acute Human Intoxication: Neurotoxin Shellfish Poisoning

The diagnosis of NSP is based upon clinical presentation and history of bivalve shellfish consumption from a risk area. Symptoms begin 15 min to 3 h after exposure. In most cases, time to illness is about 3 to 18 h but may last for several days [118]. NSP is characterized by neurological as well as gastrointestinal symptoms (nausea, vomiting, abdominal pain and diarrhea). Symptoms of greater concern to most individuals include paresthesias of the mouth, lips and tongue; peripheral tingling, partial limb paralysis, slurred speech, dizziness, ataxia, myalgias, vertigo, overall fatigue, a general loss of coordination and coma in severe cases [119]. Reversal of hot and cold temperature sensation, bradycardia, hypotension and mydriasis have also been reported. NSP is not fatal, however, hospitalizations are sometimes necessary [114,116].

Toxicokinetic studies for BTX-group toxins following oral administration in rats found that they are rapidly absorbed and widely distributed to all organs with the highest concentration found in liver where they are metabolized [120]. Given their lipid solubility, BTXs pass the blood-brain barrier [114]. Elimination is approximately equally distributed between urine and feces [14]. BTXs metabolites have been found in human urine samples collected within hours post ingestion, but they are absent in samples 3–4 days later. BTX appears to have a short half-life in serum, but the total body clearance is estimated to take days [114].

Humans may also be affected from exposure to aerosols on or near to marine waters where algal blooms have developed. Aerosolization of brevetoxins in sea spray by coastal wind and waves occurs as the dinoflagellate cells are broken down releasing the toxins into the water and subsequently into the atmosphere.

Exposure to brevetoxins could be by inhalation in humans during recreational or occupational activities and is less effectively prevented than shellfish consumption. Wildlife (fish, birds and marine mammals) is commonly exposed to aerosolized and/or ingested brevetoxins during the red tides; representing a good sentinel for human health. It is also a source of data for human risk assessment and pathological studies [14]. Toxic aerosols can cause a transient self-resolving inhalation syndrome characterized by respiratory problems and eye irritation [121]. Symptoms including sneezing, rhinorrhea and throat irritation begin minutes to hours (<24 h) after exposure. This anatomical localization of symptoms suggests a deposition of BTX in the upper airway. However, adverse respiratory effects reported during exposure to high BTXs levels include severe bronchoconstriction, mucosal irritation, cough and exacerbation of symptoms in people with asthma, which reflect the deposition of fine particles in the lower respiratory tract with subsequent irritant effects [122]. Healthy subjects rapidly reversed the respiratory disturbances by leaving the contaminated area, but in asthmatics these alterations may persist for several days [91]. In any case, most patients recover within 2 to 3 days without long term or chronic effects [114].

Toxicokinetics studies in rats indicated that by inhalation the toxin was rapidly absorbed from the lung to the blood and distributed to all tissues. The majority of BTX was cleared rapidly from lung, liver and kidneys [123].

Depending on the exposure route and presenting symptoms, treatment of intoxication may include removing the patient from exposure to brevetoxins, airway management, bronchodilators, fluid replacement, and general supportive care. Administration of sedatives and pain mitigation are the main tasks as there is no current specific antidote available for NSP [114]. Gastrointestinal decontamination with activated charcoal for patients presenting within the first 4 h post ingestion has been recommended [116]. The discovery of the natural antagonist of BTX known as brevenal, produced by *K. brevis*, may also prove to have therapeutic value in treatments of NSP [124].

FAO estimated that 2–3 µg BTX equivalents/kg b.w. is toxic in humans, but the available data on human intoxications do not allow the establishment of an oral ARfD for BTX-group toxins [14,125].

### 5.2. Chronic Toxicity of Brevetoxins

Mammalian studies have explored acute toxicity, therefore, little is known about the effects of chronic and subchronic exposure to brevetoxins [113,119]. In addition to the neurotoxic actions, brevetoxins are respiratory irritants that elicit an inflammatory response [91,126]. However, the persistence of respiratory disturbances after BTXs inhalation, which affects only asthmatic subjects, may involve neuroimmune interactions.

There are several indications of chromosomal aberrations and DNA damage induced by BTX-group toxins in vitro and in vivo, particularly BTX-2. However, neither BTX-2 nor BTX-6 were mutagenic in a reverse mutation assay [113].

### 5.3. Relative Toxic Potency of Brevetoxin Analogs

**Figure 3:**
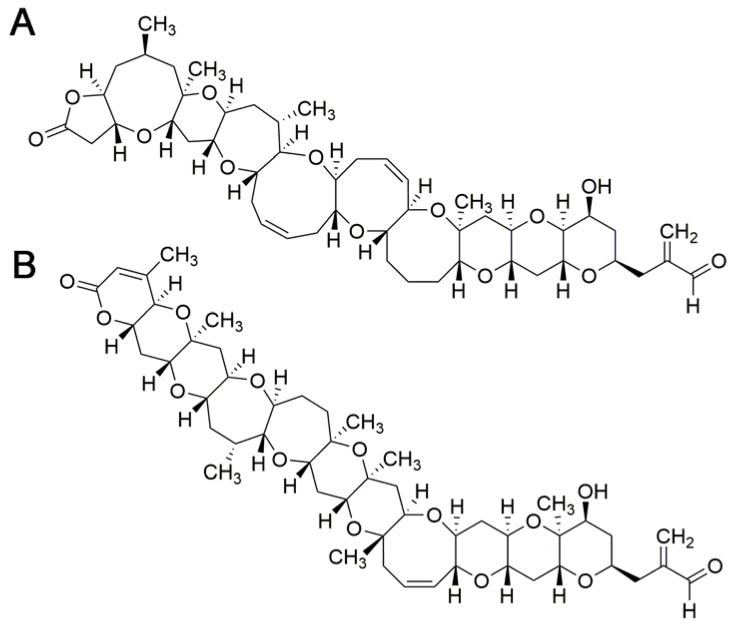
Chemical structure of brevetoxin 1 (**A**) and brevetoxin 2 (**B**).

In the BTXs group some analogs and metabolites have been identified [114]. Based on their molecular backbone structures, two types of BTX-group toxins can be differentiated (type 1 and type 2). BTX-1 and BTX-2 (Figure 3) are considered to be the parent toxins from which other BTXs derive [113]. Acute toxicity of BTX analogues and metabolites has been determined in mice by i.p. administration. BTX-2, BTX-3 and BTX-B2 appear to have similar toxic potencies. BTX-B1 is the most toxic and BTX-B4 is threefold more toxic than BTX-B2 [127]. Other BTX analogues could not be evaluated due to a lack of adequate data [113]. Oral toxicity of BTX-3 is 10 times higher than that of BTX-2 [112]. This different toxicity may be related to the absorption rate of the analogues.

Mouse bioassay (MBA) is the CODEX Standard method for BTXs and the regulatory limit is expressed as mouse units (MU). A maximum level for BTX-group toxins in shellfish is 20 MU/100 g in US, New Zealand and Australia (1 MU = 4.0 µg PbTx-2) [114]. Nevertheless, there are currently no regulations on BTX-group toxins in shellfish or fish in Europe since they have not been reported in this continent [113]. As no analytical method is used, TEFs for BTXs seem unnecessary and are not currently proposed [20].

### 5.4. Limitations and Implications of Current Toxicological Information on Brevetoxins

There are limited data on acute toxicity in animals and insufficient quantitative data on human illness attributed to BTXs. Further information is needed to better characterize the oral toxicity of BTXs and their relative potencies with emphasis on human exposures. There are no long-term studies on BTXs that would allow establishing a TDI. Confirmed NSP cases should be followed over a long period of time to determine whether there are long term adverse effects or chronic sequels to exposures.

Monitoring BTX levels in shellfish during harmful algal blooms (HABs) maintains a low NSP incidence [14]. The apparent trend towards expansion of BTX producing algae suggests that those toxins could also emerge in Europe for instance. Without regulatory limits set in EU legislation, some potential prevention measures would be to adopt the regulation utilized in other regions such as US. Monitoring of coastal water and seafood together with clear, easily available information on recreational closures could decrease the risk of NSP. Besides, persons with asthma or other respiratory problems should avoid beaches during “red tides”.

## 6. Tetrodotoxins

Tetrodotoxin (TTX, Figure 1C) and its 30 analogues are potent neurotoxins produced by bacteria *Pseudomonas* and *Vibrio* among others [128]. TTX poisoning has been reported worldwide. It was traditionally associated with consumption of pufferfish in Japan [129], but was also found in other marine animals including, goby fish, gastropods, crabs or bivalves in countries such as Australia, Bangladesh, Brazil, China, Israel, Morocco, Singapore, Taiwan and USA. Recently, TTXs have been found in gastropods and marine bivalves along the European Coasts (Spain, Greece, UK and The Netherlands) [130,131].

TTX and analogues bind to site 1 of the voltage-gated sodium channels (Na_v_) and prevent access of monovalent cations to the sodium channel pore. TTX affects both action potential generation and impulse transmission, resulting in a blockade of the nerve conduction and in muscle paralysis [132,133].

### 6.1. Acute Human Intoxication

TTX and analogues are acutely toxic in humans. TTX toxicity is caused by the inhibition of nerve and muscle conduction. This potent neurotoxin affects skeletal muscles and tissues in the digestive tract, the diaphragm and the respiratory system. Symptoms of toxicity can occur within 10–45 min of ingestion, although a delayed response of 3–6 h has also been reported [134]. Patients with acute poisoning usually recover without residual deficits although some take a few days to recover. The sequence of acute symptoms are: perioral numbness and paraesthesia with or without gastrointestinal symptoms; lingual numbness, early motor paralysis, incoordination, slurred speech with normal reflexes; generalized flaccid paralysis, aphonia and fixed/dilated pupils; severe respiratory failure and hypoxia, hypotension, bradycardia, cardiac dysrhythmias and unconsciousness. In fatal cases, death is caused by respiratory failure and cardiac collapse [135].

There is no specific treatment for TTX since there is no antidote against TTX poisoning. All patients receive supportive treatment. Other treatments are based on responding to manifested signs and symptoms. Removal of unabsorbed toxin may be attempted by induced vomiting or by gastric lavage giving activated charcoal to bind to the toxin. Instillation of 2% sodium bicarbonate has been suggested since TTX is less stable in an alkaline environment. Other treatment options included cysteine, cholinesterase inhibitors, naloxone and steroids [129].

There is limited information about toxicokinetics of TTXs. However, it seems to be rapidly absorbed in the human digestive tract, based on the short delay between ingestion and the onset of the symptoms. Data from human poisoning cases showed that TTX could be detected in the urine after a few hours and up to 7 days after the ingestion of contaminated fish [136]. Plasma/serum concentrations of TTX fall rapidly and may be undetectable after 6–24 h [137]. No data are available regarding the distribution and metabolism of TTX in humans, and little is known in animals [138].

The doses at which TTX causes acute toxicity and lethality in humans are unclear; there are some human case reports that indicate that acute poisoning can result from doses of 4–42 µg/kg b.w. Therefore, CONTAM Panel decided to derive an ARfD based on the animal data, considering human data and data on STX as supportive information. ARfD of 0.25 µg/kg b.w. was derived from a TTX dose of 25 µg/kg b.w., at which no adverse effects were observed in an acute oral study with mice, applying a standard uncertainty factor of 100 [138]. This ARfD (0.25 µg/kg b.w.) is 16-fold lower than the lowest dose at which severe effects have been observed in humans (4 µg/kg b.w.).

### 6.2. Chronic Toxicity of Tetrodotoxins

No data on long-term effects of TTX have been identified. There is a lack of subchronic or chronic studies on TTX in animals. TTX did not show any genotoxic activity in a battery of good laboratory practice (GLP)-compliant in vitro and in vivo assays conducted according to the Organization for Economic Co-operation and Development (OECD) guidelines [128].

### 6.3. Relative Toxic Potency of Tetrodotoxin Analogs

Mouse bioassay is the most widely applied detection method for TTXs outside the EU. However, this test does not distinguish between TTX and its analogues and TTX from STX. TTXs can be detected as different compounds and quantified using analytical methods such as liquid chromatography with mass spectroscopy (LC-MS). In this case, TEF should be applied to determine the total content of TTXs [128].

The potencies for TTX analogues are lower than that of TTX (they seem at least 5- to 100-fold less toxic than TTX). Therefore, the ARfD 0.25 µg/kg b.w. established to TTX could be applied to TTX analogues detected in marine bivalves and gastropods (TTX, 11-oxoTTX, 4-epi-TTX, 4,9-anhydro-TTX, 5,6,11-tri-deoxy-TTX and 11-norTTX-6-ol). Nevertheless, more data obtained after oral exposure are needed to estimate relative potencies of TTX analogues [60].

### 6.4. Limitations and Implications of Current Toxicological Information on Tetrodotoxin

European Regulations established that live bivalve molluscs placed on the market for human consumption must not exceed limits for marine biotoxins, but no limits are currently listed for TTXs. However, fishery products derived from poisonous fish of the families Tetraodontidae, Molidae, Diodontidae and Canthigasteridae should not be marketed [128].

There is limited information on the occurrence of TTXs in edible parts of marine gastropods and bivalves, and scarce consumption data are available for marine gastropods. However, EFSA CONTAM Panel on Contaminants in the Food Chain states that a concentration below 44 µg TTX equivalents/kg shellfish meat is expected not to result in adverse effects in humans [128]. This value is based on a large portion size of 400 g for bivalves, an adult body weight of 70 kg and a group ARfD of 0.25 µg TTX/kg b.w.

TTX is heat stable and will not decompose during the traditional cooking process [27,131]. Since TTX and its analogues are chemically similar, it can be assumed that they will behave in the same way. However, more data on TTX and its analogues should be obtained during cooking or freezing to provide a reliable exposure assessment.

There is also a need for further information on toxicokinetic as well as oral toxicity of TTX and its analogues. Adequate studies are required to estimate the relative potencies of TTXs analogues after oral exposure. Besides, TTXs chronic effects should be explored, taking also into account that TTX is now considered a therapeutic molecule to use in certain pathologies involving Na_v_ channels, particularly in the field of pain including chronic pain conditions [139].

## 7. Okadaic Acid Group

Okadaic acid (OA) and dinophysistoxins (DTXs) are lipophilic heat-stable toxins produced by marine dinoflagellates, most notably *Dinophysis* and *Prorocentrum* [140]. They accumulate in filter-feeding shellfish (mussels, oysters, scallops, clams, cockles) and may cause the gastrointestinal syndrome diarrhetic shellfish poisoning (DSP) in humans by the ingestion of contaminated seafood. DSP have been reported worldwide in the US, South America, Europe, Canada, New Zealand and Japan [57,141].

**Figure 4:**
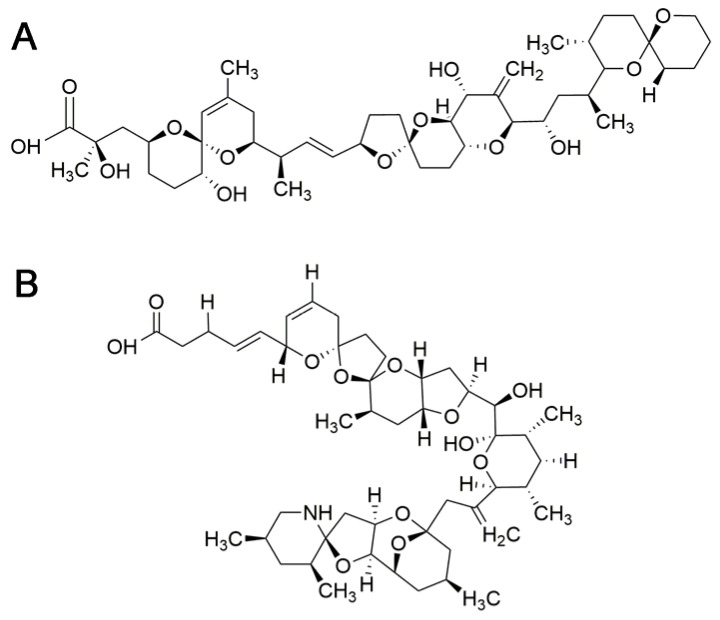
Chemical structure of okadaic acid (**A**) and azaspiracid-1 (**B**).

OA is the reference compound of DSP toxins (Figure 4A). Dinophysistoxin 1 (DTX1) and dinophysistoxin 2 (DTX2) are also main analogues associated with diarrheic episodes. In addition, in shellfish there are different esters of OA and DTXs referred to as dinophysistoxin 3 (DTX3) that are almost non-toxic [142] but may be hydrolyzed after ingestion in the gastrointestinal tract and release the parent compound (OA, DTX1, or DTX2) [142,143].

DSP toxins are powerful protein phosphatase (PP) inhibitors. The main target is protein phosphatase 2A (PP2A) and the second targets are protein phosphatase 1 (PP1) and protein phosphatase 2B (PP2B) [144,145]. Phosphatases are important modulators of cell signaling pathways and their inhibition may result, for instance, in loss of control over sodium secretion and solute permeability in the cell [145]. However, the association of diarrhea with protein phosphatase inhibition is not established, since other potent phosphatase inhibitors do not show diarrheic effect [146,147]. Therefore, other actions could be involved in the toxicity of OA and DTXs, for instance inhibition of the neuropeptide Y that protects against diarrhea by inhibition of intestinal motility and water and electrolyte secretion [148].

### 7.1. Acute Human Intoxication: Diarrheic Shellfish Poisoning

Diagnosis of DSP is made by dietary history and symptoms that begin from 30 min to 5 h after eating toxic shellfish. The main symptom is incapacitating diarrhea, but DSP is also characterized by symptoms such as gastrointestinal distress, nausea, vomiting, and abdominal pain, as well as sometimes headache, chills and fever [149]. The symptoms may be severe, continue for about 2–3 days and lead to dehydration, which is an important health problem. However, this syndrome is usually self-limiting with full recovery and no fatalities have been reported [150].

There is no antidote, with this in mind, treatment of DSP includes replacement of electrolyte and fluid loss.

In risk assessment, due to the lipophilic nature of DSP toxins and previous studies, their bioavailability has been considered equal to the amount of toxins present in the food. However, the absorption of OA through the human gut barrier has been studied using in vitro intestinal Caco-2 cell models showing a poor permeability to the intestinal tract [151]. Moreover, a recent study reported that bioaccessibility of OA total content was 88% in mussels and 75% in donax clams [142]. Therefore, it was suggested that DSP toxins might exert their toxic effects in the digestive tract before entering the blood stream [142,152].

There are no quantitative data on OA toxicokinetics in humans, but DTX3 could be converted to the parent compounds in the gastrointestinal tract [153]. In mice, OA oral doses by gavage were absorbed by the gastrointestinal tract, distributed to all internal organs and slowly eliminated in urine and feces [154]. Recent experiments conducted in mice indicated that 24 h after oral administration, concentrations of DTX2 in urine were higher than in feces [155]. The elimination pattern indicated biliary excretion and enterohepatic circulation. Besides, studies in pregnant mice demonstrated that OA might pass the placental barrier [156].

The Joint FAO/IOC/WHO Expert Consultation on Biotoxins and Bivalve Molluscs established in 2004 a provisional ARfD value of 0.33 µg/kg b.w. [53]. This ARfD is consistent with the 0.3 µg/kg b.w. proposed in 2007 by the EFSA CONTAM Panel on marine toxins, based on the effects in a large number of affected shellfish consumers from various countries and comprising also the most sensitive individuals [157]. The Panel concluded that a LOAEL for human illness is 50 µg OA equivalents/person, which approximates to 0.8 µg OA equivalents/kg b.w. An uncertainty factor of three was applied to extrapolate this LOAEL to a NOAEL yielding an ARfD of 0.3 µg OA equivalents/kg b.w. [27].

### 7.2. Chronic Toxicity of Okadaic Acid

OA has shown some evidence for genotoxicity in non-standard in vitro assays but for DTX2 and DTX3 no genotoxicity data are available [158]. The genotoxic and cytotoxic effects induced by OA if left unrepaired or inaccurately repaired cause genomic instability that may lead to severe pathologies including cancer [159]. In vivo studies indicate that chronic exposure to OA may promote tumor [140], and both OA and DTX1 showed a tumor promoting activity in a two-stage carcinogenicity study in mice [157,160]. One proposed mechanism to explain the role of DSP toxins as tumor promoters involves an increase in the synthesis and secretion of TNF-α [161]. It was recently demonstrated that OA and DTX1 at sublethal doses stimulate secretion of inflammatory factors [162]. Furthermore, sublethal doses of OA can increase cellular mitosis in vivo and activate events involved in the inflammatory response and carcinogenesis [163]. It has also been suggested that chronic exposure to DSP toxins may increase the risk of gastrointestinal cancer [164]. In humans, an association between DSP and stomach and pancreas cancer in females; and esophagus, stomach, colon and liver cancer in males, was first described by Cordier et al. in 2000 [165]. Lopez-Rodas et al., in 2006 obtained correlations between the consumption of shellfish and the total incidence of tumors [166]. However, there is scarce data on the chronic toxicity of OA and DTXs in humans to reach a definite conclusion.

### 7.3. Relative Toxic Potency of Okadaic Acid Analogs

In order to protect consumers, the regulatory authorities have defined the maximum level of DSP toxins as 160 µg of OA equivalent per kg of shellfish meat for human consumption [32] and the European Commission has established the liquid chromatography mass spectrometry (LC-MS) as the reference method for their detection [167]. However, quantitation of the toxin analogues is not enough for monitoring since the content of toxins is expressed as the sum of OA equivalents and different analogues may have dissimilar toxic potencies. Therefore, determination of TEFs is required [20]. For monitoring purposes and shellfisheries management, each toxin quantified in the shellfish matrix is multiplied by its TEF. If their sum results in values above the regulatory limit, the sample is considered unsafe for human consumption.

OA and DTX1 showed similar toxicity in mice when administered i.p., while potency of DTX2 was lower [20,168,169]. Based on the information on the i.p. toxicity, EFSA established the following TEF values: OA = 1, DTX1 = 1, DTX2 = 0.6 [157]. Since DTX3 can be hydrolyzed in the gut, TEF values for this toxin can be considered equal to those of the corresponding unesterified toxins (OA, DTX1 and DTX2) [60]. However, DSP is due to ingestion of shellfish contaminated by OA and analogues; therefore, TEF should be established on the basis of their acute oral toxicity [170]. Due to the lack of appropriate information about human intoxications, oral toxicity in animals has been recognized as the most relevant for updating TEF [27]. There are discrepancies in the reported oral lethal doses of OA and analogues due to the variation in the quality of the toxins used in the toxicological studies [60,171]. However, in all cases the diarrhetic effect is very fast even in animals that do not suffer damage to the intestinal mucosa and do not die [155,172]. A recent in vivo comparative study following the guidelines issued by the OECD and using certified standards of toxins determined the oral LD_50_ of DSP toxins and concluded that DTX1 is more toxic than OA while DTX2 showed lower toxicity than OA [152,155]. Data of this study allowed an estimation of the TEFs for OA and analogues based on oral toxicity suggesting a re-evaluation of the current TEF in order to determine the actual risk to seafood consumers [60].

### 7.4. Limitations and Implications of Current Toxicological Data

There is little information that links the inhibition of PPs caused by OA and analogues and diarrhea. More studies are required to confirm and clarify the DSP actions that trigger their acute toxic effects.

No tolerable daily intake could be established due to the lack of data on chronic toxicity of DSP toxins in animals or humans [27]. Further studies about chronic toxicity of DSP toxins are needed in order to establish the potential risk for human health due to the continuous presence of these toxins in seafood [53].

Blooms of dinoflagellates related with DSP are difficult to control but their impact will grow in parallel with aquaculture expansion. Therefore, monitoring seafood is important to ensure that products placed on the market do not exceed the limit of 160 µg OA/kg helping DSP prevention [57,173].

Toxin regulatory limits have been established in shellfish meat, but there are scarce data of the possible impact of the processing of shellfish in their toxicity. Actually, in 2015, the EU-Harmonised Standard Operating Procedure for Determination of Lipophilic Marine Biotoxins in Molluscs included an annex providing details on the procedure for the extraction of these toxins from processed mussels in order to take into account the loss of water due to steaming and or autoclaving [174]. Therefore, the effect of cooking or freezing of seafood that has been naturally exposed to DSP toxins should be better studied.

## 8. Azaspiracids

Azaspiracids are polyether compounds described after a gastrointestinal poisoning outbreak in 1995. This class of toxins comprises more than 40 analogs with different toxic potencies [175,176]. The representative molecule is AZA1 (Figure 4B). Parental compounds are produced by dinoflagellates of the genera *Azadinium* and *Amphidoma* [177]. Bioconversion of phytoplanktonic parental compounds in shellfish contribute to the high number of azaspiracid analogs. These toxins have a global distribution, although they have been more frequently described in temperate waters [177,178,179,180,181,182,183,184,185,186,187]. Azaspiracids accumulate in other marine organisms following the trophic web, mostly in filter feeding molluscs including mussels, oysters, scallops, clams or cockles [182,188,189,190].

The biological target of this toxin class is still unknown.

### 8.1. Acute Human Intoxication: Azaspiracid Poisoning

Azaspiracids reach humans through ingestion of contaminated shellfish and cause azaspiracid poisoning (AZP). Several AZP episodes have been reported in the last 25 years in Europe and the United States [183,188,191,192,193,194,195,196]. Humans exposed to AZAs displayed gastrointestinal symptoms such as nausea, vomiting, diarrhea and stomach cramps [184,188]. Headache has also been reported [193]. The symptoms appear around 3 h after ingestion and last for about 15 h [193]. Complete recovery occurs within 2–5 days [193]. Treatment consists of supportive therapy, mainly fluid and electrolyte replacement.

There are no pharmacokinetics data in humans. Intestinal absorption has been demonstrated in vivo in mice and in vitro in human intestinal cell cultures [197,198]. AZA1 is widely distributed after absorption, being detected in several organs including spleen, kidney, lung, heart and liver, and only trace amounts in the brain [198]. AZA1 amount is reduced in all organs, except the kidneys, 7 days after acute exposure.

The most recent estimation of AZAs LOAEL is 1.9 µg AZA1 equivalents/kg b.w. [192], which is based on the scarce data collected from human intoxications. The NOAEL was derived from the LOAEL considering uncertainty factors of 3 to account for extrapolation from NOAEL and 3 for the small number of individuals. The ARfD was established at 0.2 µg AZA1 equivalents/kg b.w. [192]. These estimates differ from the LOAEL and ARfD proposed by the joint FAO/WHO/IOC report, with values of 0.4 and 0.04 µg/kg respectively, owed to the application of different safety factors to account for uncertainties [14].

### 8.2. Azaspiracid Chronic Toxicity

Chronic toxicity of AZAs to humans is unknown. The regulatory limits of AZAs in shellfish meat are based on acute toxic effects. However, the effects of repeated exposure to subacute doses of these toxins should also be considered. Actually, the toxicity related to AZAs may last in shellfish for months [193,199], and there have been many reports of levels of azaspiracids below regulatory limits in different locations [177,179,180,182,190,200,201,202].

Some evidence of a potential chronic risk has been obtained in animal studies. In mice, repeated oral exposure to sublethal doses of AZA1 (1 and 5 µg/kg, 40 times, twice a week) consistently caused erosion of the small intestine in all individuals (shortened and damaged microvilli) [203]. Lung tumors were also reported in mice that received higher amounts of AZA with an incidence of 10% and 30% at doses that caused 90% and 30% mortality, respectively. However, recent in vitro studies in mammalian cell cultures indicate that AZAs are not genotoxic [204]. It should be noted that these early toxicological studies were not performed with certified toxin standards [203]. AZA1 has also been found to have cardiotoxic potential in rats. After repeated i.p. administration of sublethal doses, the rats displayed signs of heart failure and alteration of myocardium structure, as well as increased molecular markers of cardiomyocyte damage in heart tissue (1 and 10 µg/kg, 4 doses, 4 days interval), without signs of toxicity in other organs [205,206].

### 8.3. Relative Toxic Potency of Azaspiracids

The information about AZAs acute toxicity to humans has been estimated from samples containing several analogs; therefore, the toxic potency of the different AZA analogs in humans is unknown. Currently, the estimation of AZA-related shellfish toxicity is based on the quantification of at least AZA1-3 [167], and the TEFs extrapolated from mouse i.p. LD_50_ of these three molecules, with values of 1.0, 1.8 and 1.4 for AZA1, AZA2 and AZA3, respectively; AZA1 being the reference compound [192]. Very recently, more relevant TEFs have been proposed from oral LD_50_ results in mice in a joint FAO/WHO technical paper [27,207]. The oral (gavage) toxicity-based TEFs would be 1.0, 0.7 and 0.5 for AZA1, AZA2 and AZA3 respectively. In addition, a TEF value of 0.7 for AZA6 has been proposed from its i.p. toxicity in the FAO/WHO technical paper on TEFs [27].

### 8.4. Limitations and Implications of Current Azaspiracid Toxicity Data

Evidence of the threat that AZAs pose to human health has prompted the regulation of a maximum limit of AZAs in shellfish destined for human consumption in several countries. As for most toxin groups, the scarcity of chronic toxicity information precludes the estimation of a TDI. Therefore, current limits of AZAs are based on ARfD [192]. Legal regulations and Codex Alimentarius provisions fix a maximum limit of AZAs content in shellfish at 0.16 mg of azaspiracid eq/kg of flesh [31,32].

Currently, detection of AZAs is performed mainly by LC-MS [167]. Analytical methods rely on TEF values of the different analogs of the group for an adequate estimation of sample toxicity. In spite of the recently available data on oral toxicity, these studies were performed only with AZAs 1–3. There is no information of oral toxicity of other AZA analogs. The scarce amount of most analogs has hampered toxicological studies in vivo, even by i.p. administration, which require lower quantities of toxin. Intraperitoneal LD_50_ values are available only for AZAs 1–3 and 6 [27].

Bioconversion of parental compounds and possible impact of the processing of shellfish in AZAs toxicity should be further studied. It has been shown that AZA17 and AZA19 are main products of bioconversion from AZA1 and AZA2 by oxidation of the methyl-group at C22 in mussels [208]. Furthermore, decarboxylation of these products induced by heat treatments to produce AZA3 and AZA6, respectively, was also described, which suggest that these analogs should be considered for estimating the toxicity risk [209].

Another point that should be discussed is that no mice showed signs of diarrhea after oral administration in these experiments, which suggests that AZAs toxicity may be different in this species and in humans. Therefore, although more meaningful than the i.p. TEFs, oral TEFs obtained in mice may not be a perfect reflection of the relative potency of AZAs for gastrointestinal toxicity in people.

With regards to chronic toxic effects, there is no information whatsoever about toxicity to humans. The chronic or subacute toxicity data available at the moment have been obtained in rodents. In addition, cardiotoxicity was detected after i.p. administration, and, although there is evidence of AZAs absorption after oral administration [207], the cardiotoxic effect should be confirmed by the oral route. In general, chronic/subacute effects should be tested also in other species, considering the differences found between humans and rodents regarding toxic symptoms.

## 9. Palytoxin

**Figure 5:**
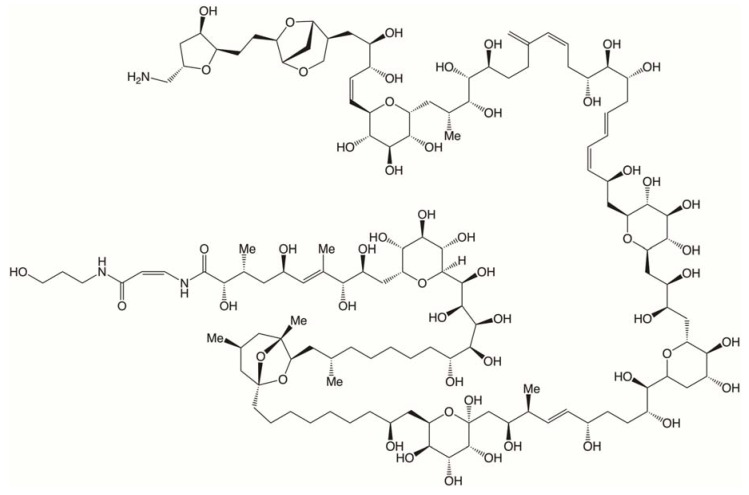
Chemical structure of palytoxin.

Palytoxins (PLTXs) are the biggest non-polymeric molecules of natural origin. With molecular weights in the order of 2700 Da, palytoxins have one of the more complex structures described to date (Figure 5). More than 25 analogs have been reported including PLTX, 42-hydroxy PLTX (2 isomers), homoPLTX, bishomoPLTX, deoxiPLTX, neoPLTX, iso PLTX, isobaricPLTX, ovatoxins-a to -k, ostreocins-B and -D, and mascarenotoxins-a and -b [210,211,212].

Palytoxins are produced by soft corals of the genera *Palythoa*, *Zoanthus* and *Parazoanthus*, by planktonic and benthic dinoflagellates of the genus *Ostreopsis*, and by cyanobacteria of the genus *Trichodesmium* [210]. Although initially palytoxins were described in corals from tropical waters, currently this toxin class is distributed worldwide. Most episodes of PLTX-related seafood poisoning have occurred in tropical and subtropical areas; however, global distribution of *Ostreopsis* and the trade of soft corals for aquarium decoration purposes contribute to the global distribution of palytoxin-related toxicity reports by other exposure routes [213].

The presence of these toxins has been described in several marine animals at different levels of the trophic web, among them, several fish species, sea urchin, molluscs, crabs and octopus [214,215,216,217,218]. 

The toxicity of PLTX has been attributed mainly to its action on the Na^+^/K^+^-ATPase pump, which is critical to maintain membrane polarity. Palytoxin disrupts the function of the Na^+^/K^+^ pump, that upon binding of the toxin behaves as a cationic channel [219]. In vitro toxicity studies suggest that ostreocin-D shares the same mechanism of action [220,221].

### 9.1. Human Acute Toxicity of Palytoxins

Toxicity of palytoxins in humans has been reported after exposure through ingestion, inhalation and dermal and corneal contact. The consequences of palytoxin toxicity may be life threatening mainly by ingestion or inhalation [222,223,224,225]. A few cases of intoxication due to ingestion of PLTX-contaminated seafood (fish or crustaceans) in tropical and subtropical areas have been published, some of them with a fatal endpoint [210,222,226,227]. However, occurrence may be higher than reported because confirmation of PLTX presence in the leftovers is not usually performed after foodborne intoxication. The symptoms by this route include bitter taste, oral and limb numbness, nausea, dizziness, abdominal pain, myalgia and arthralgia, progressing to cardiovascular disfunction with dysrhythmias, electrolyte imbalance, dyspnea and death [222,227,228,229]. Rabdomyolysis is considered a characteristic of PLTX toxicity to humans based on elevated plasma levels of creatine kinase and myoglobinuria detected in PLTX intoxication victims [226]. 

In the last two decades, several reports of suspected or confirmed toxicity of palytoxin by inhalation and dermal contact have been published. PLTX-related toxicity has been reported after inhalation of steam generated by cleaning of *Palythoa*-containing aquariums with hot water [223,224,225,230,231,232,233]. Similar symptoms have been described after inhalation of aerosols during *Ostreopsis* blooms by beachgoers [234,235,236,237]; with ovatoxins being the main PLTX-analogs present in this kind of blooms [237,238]. Symptoms by inhalation exposure consist of fever, breathing difficulty, runny nose, cough, and weakness and myalgia among others [223,224,225,230,231,232,234,235,236]. Hospitalization for 2–3 days is necessary in cases of acute respiratory failure [223,224,225]. Recovery is slow in severe cases considering that dyspnea and fatigue were reported in some patients for 1–3 months after exposure [224]. Skin contact with corals and *Ostreopsis* containing aerosols or water also cause signs of dermal toxicity such as dermatitis, erythema and pruritus [232,239].

Corneal toxicity has appeared after manipulation of soft corals without gloves or goggles. The patients display keratoconjuntivitis, eye pain, photophobia and decreased vision. In more serious cases, corneal ulcer, dyspnea, nausea and shivering may occur [240,241,242].

PLTX toxicokinetics in humans has not been studied, and the data in animals are very scarce. PLTX and ostreocin D cause tissue damage in several organs including lung, kidney, and gastrointestinal tract by oral, intratraqueal and sublingual administration in rodents [243,244], suggesting that absorption does occur through these routes. The reports of systemic symptoms in human patients after ocular exposure to PLTX also suggest that absorption may occur through routes different from ingestion in humans [242].

There are no reliable reports that would allow deriving an ARfD in humans [245]. Therefore, an estimation of ARfD has been done from two toxicological studies in animals that reported NOAEL and LOAEL values of 300 and 200 µg/kg, respectively, after PLTX administration by gavage [243,244]. LOAEL of ostreocin D was estimated at 300 µg/kg [244]. Uncertainty factors of 10 accounting for intraspecies variation, 10 for interspecies variation and 10 for the apparently lower sensitivity of mice and the possible absorption through oral mucosa were applied to obtain an ARfD value of 0.2 µg/kg b.w. for the sum of PLTX and ostreocin D [245].

Although PLTX is toxic through several exposure routes, an ARfD has not been proposed for routes other than oral. Palytoxin is actually one of the most toxic compounds by parenteral administration (i.v. LD_50_: range from 0.025 µg/kg in rabbits to 0.15–0.53 µg/kg in mice, i.p. LD_50_: 0.31–1.5 µg/kg in mice and 0.63 µg/kg in rats) [246]. Remarkable intratracheal toxicity was also reported in rats with LD_50_ values of 0.18 µg/kg [247].

### 9.2. Palytoxin Chronic Toxicity

There is currently no information about chronic toxicity of palytoxins in humans. In vivo experiments of repeated i.p. administration of PLTX for 6 weeks demonstrated alteration of lymphoid tissues, with a reduction of thymus and an increase of spleen weights. A decrease of lymphocytes in blood and tissues was also observed [248]. The animals recovered after 1 month of toxin treatment interruption. Recently, PLTX toxicity after repeated oral administrations by gavage for one week to mice was explored. This treatment protocol was lethal at doses as low as 30 µg/kg of PLTX once daily. In this study, mice that received an oral dose of 3 µg/kg/day for 7 days showed no effect [249]. The small amount of pure PLTXs worldwide has limited in vivo experiments of repeated administrations with adequate numbers of animals. In vitro and in vivo evidence suggests that palytoxin can act as a tumor promoter when combined with certain initiators; however, it is not an initiator or mutagenic [246]. Palytoxin has been identified as a tumor promoter in the multi-stage mouse skin assay [250,251], although it does not have initiator activity [246,250]. Evidence of tumorigenic or carcinogenic activity in humans is lacking.

### 9.3. Relative Toxic Potency of Palytoxins

There is no information about toxic potency of these toxins in humans. From animal studies, LD_50_ values are usually compared to derive relative toxic potencies, and as indicated before, for food safety purposes, oral LD_50_ would be preferable to other administration routes. PLTX oral LD_50_ has been estimated at 510–767 µg/kg [243,252]. With regards to other members of the PLTX class, oral LD_50_ is only available for 42-hydroxyPLTX, whose potency is similar to PLTX with a value of 652 µg/kg [253]. For ostreocin-D toxicity, evaluation was performed by i.p. injection in mice with an LD_50_ of 0.75 µg/kg, that was deemed similar to PLTX i.p. LD_50_ (most estimations in the range 0.32–0.72 µg/kg) [245,246,254]. Similar toxicities of PLTX and ostreocin-D were also reported in vitro for a neuroblastoma cell line [220], although in other cell types, the ostreocin-D toxic effect was lower [246]. Recent in vivo studies report a lower toxic potency of ostreocin-D by several administration routes, although LD_50_ values are not provided [244]. Only in vitro studies are available for ovatoxin-a, which has shown 100-fold less toxic potency than PLTX in a skin keratinocyte cell line [255].

### 9.4. Limitations and Implications of Current Palytoxin Toxicity Data

Although the threat to human health coming from marine toxins has been usually associated to food, it is clear now that palytoxins have important toxic effects through other routes. The real prevalence of PLTX-related intoxications is unknown [232], and therefore, the health risk related to this toxin class has probably been underestimated. Although the EFSA panel proposed a maximum permitted level of 30 µg/kg of palytoxin and ostreocin-D in seafood based on ARfD values [245]; currently, there is no specific regulation of PLTX levels in seafood, mostly due to the scarce information on PLTX occurrence, which impairs adequate risk assessment [245].

In the absence of quantitative data regarding PLTX toxicity to humans, toxicological data obtained in animals have been used for an estimation of ARfD; however, this is far from optimal. The data used for oral ARfD estimation were obtained using oral gavage administration [243,252], although FAO/WHO recommendation is to use voluntary ingestion for regulatory purposes [27]. In another study, no sing of toxicity was displayed at doses of 2500 µg/kg after voluntary ingestion [252], but due to the lack of reference PLTX standards, a thorough description of the toxin source and analytical characteristics, which is lacking in this report, would be necessary for reliable risk evaluation. In addition, for ARfD calculation, an uncertainty factor of 10 was applied based on possible absorption through buccal mucosa, besides apparent lower mouse sensitivity; however, buccal absorption from contaminated seafood would probably be different than from a toxin solution [246]. Whereas ARfD estimation for all toxin groups have to be made assuming some uncertainties, in the case of PLTXs the degree of uncertainty is considerably higher than in other toxin groups. Collection of epidemiological data from PLTX-related toxic episodes is absolutely necessary to improve risk assessment. Quantification of PLTX in seafood or any other source of toxin will be critical to estimate ARfD or TDI values based on human toxicity. In the meanwhile, the recent results obtained in repeated oral administration experiments in mice should be considered for risk re-evaluation.

General public outreach and education of clinicians about the multiple routes of exposure that have been related to PLTX toxicity are needed to improve prevention and diagnosis. Warning of the risk of certain coral species and guidelines for the correct manipulation to minimize exposure and for culture conditions to reduce toxin production are required to protect aquarium hobbyists health. Identification of toxic coral specimens should be regulated in aquarium trading activities.

As mentioned above, the scarce information about toxic potency of these compounds comes from animal studies. Assessment of toxic potency of other analogs of the palytoxin class is largely lacking. In addition, none of the existing results were obtained with certified reference material because certified standards are not available for any member of this toxin class.

## 10. Concluding Remarks

Marine biotoxins pose a serious threat to human health (see Table 3 for a summary of symptoms of acute poisoning). Nevertheless, the toxicity mechanism of some of them remains unknown, mostly due to a lack of enough purified material for toxicity trials. Consumer exposure to an acute toxic amount has been reduced by establishment and implementation of legal regulations for many toxin groups (see Table 4 for a summary of current regulations). Intoxication routes other than oral should also be considered and emerging toxins could require regulatory limits. However, the improvement of protection plans will require in the near future the collection of more epidemiological data using rigorous systematic procedures in order to re-evaluate risk assessment. Chronic toxicity of these molecules should also be considered for human exposure hazards. Current information on chronic toxicity is very scarce for marine biotoxins, and more studies about the impacts of repeated subacute doses by oral route for long periods of time should be performed to determine the relevance of TDI-based risk evaluation and propose TDI values. Pharmacokinetics information is also very scarce for all toxin classes. With regards to the simultaneous exposure to several toxins, it is noteworthy to investigate to which mixtures of biotoxins the consumers can be exposed and to which levels, and the toxicological implication of toxin combinations. Adequate sample toxicity estimation also requires reliable TEFs for all the analogs of the toxin class obtained by oral administration and with certified toxin standards. Effective consumer protection related to marine toxins depends greatly on the toxicological information available for these compounds. Finally, the number of marine toxins with human toxicity will probably keep increasing in the future considering the continuous description of toxic syndromes related to consumption of marine animals, such as chelonitoxism due to the ingestion of sea turtles for which the causative agent has not been unequivocally identified yet [256,257,258].

## Figures and Tables

**Table 1:** Oral toxicity and TEF values proposed for PST toxins.

Toxin	TEF, 2009 EFSA	Oral LD_50_ Gavage (nmol/kg)	Oral LD_50_ Feeding (nmol/kg)	Relative Toxicity Gavage/Feeding	TEF, 2016 FAO/WHO
STX	1	1190	3200	1	1
NeoSTX	1	700	1260	1.7/2.54	2
GTX1	1				1
GTX4	0.7				0.7
GTX1&4		1610	3420	0.74/0.93	
GTX2	0.4				0.4
GTX3	0.6				0.6
GTX2&3		2230	5590	0.53/0.57	
GTX5	0.1	18,900	50,000	0.063/0.064	0.1
GTX6	0.1	31,100	>188,000	0.038/<0.017	0.05
C1					0.01
C2	0.1				0.1
C1&2		35,000	74,000	0.034/0.043	
C3	0.1				0.01
C4	0.1				0.1
C3&4		42,700		0.028/-	
dc-STX	1	2600	8680	0.46/0.37	0.5
dc-neoSTX	0.4	5500	14,300	0.22/0.22	0.2
dc-GTX2	0.2				0.4
dc-GTX3	0.4				0.4
dc-GTX2&3		7130	29600	0.17/0.11	
References	[2]	[28,29]	[28,29]	[28,29]	[27]

**Table 2:** Mouse i.p. LD_50_ and EFSA adopted TEF values. P-CTX-1 is the reference toxin.

Ciguatoxin	Mouse i.p. LD_50_ (µg/kg b.w.)	Reference	Proposed TEF (EFSA)
P-CTX-1	0.25	[103,104]	1
P-CTX-2	0.9	[82]	0.3
P-CTX-3	0.9	[103]	0.3
P-CTX-3C	2	[82]	0.2
2,3-dihydroxy P-CTX-3C	1.8	[105]	0.1
51-hydroxy P-CTX-3C	0.27	[105]	1
P-CTX-4A	2	[106]	0.1
P-CTX-4B	4	[106,107]	0.05
C-CTX-1	3.6	[97,108]	0.1
C-CTX-2	1	[97]	0.3

**Table 3:** Seafood Intoxications.

Seafood Poisoning	Vectors	Onset Time and Duration	Major Acute Symptoms	Treatment
Paralytic shellfish poisoning (PSP)	Bivalve molluscs (clams, mussels, oysters, scallops), crab, lobster, gastropods, cephalopods, Atlantic salmon, herring, mackerel, puffer fish	Symptoms begin from 30 min to a few hours and can persist more than 24 h	Neurologic symptoms (tingling of lips and tongue, paresthesias, weakness, ataxia, dizziness, shortness of breath)	Supportive care: fluid therapy, respiratory support.
Amnesic shellfish poisoning (ASP)	Clams, mussels, oysters, scallops, squid, sardines, anchovies, crab and lobster	Symptoms begin from 15 min to 48 h and can persist several months in severe intoxications	Gastrointestinal (nausea, vomiting, diarrhea…) and neurologic symptoms (confusion, disorientation, memory loss, seizures)	Respiratory support and correction of cardiac dysrhythmias and hemodynamic instability
Ciguatera fish poisoning (CFP)	Fish, molluscs	Symptoms begin from 0.5 to 12 h. The acute phase lasts for 2–4 days. In the chronic phase symptoms persist weeks or months.	Gastrointestinal (nausea, vomiting, diarrhea…), neurologic symptoms (cold allodynia, itching, dizziness, ataxia, fatigue), cardiovascular (hypotension, bradycardia)	Intravenous mannitol and supportive care
Neurotoxin shellfish poisoning (NSP)	Mussels, clams, whelks, conch, coquinas, oysters, scallops, organs of some planktivorous fish	Symptoms begin 15 min to 3 h and last for a few days	Neurological (paresthesias, peripheral tingling, ataxia, myalgias, loss of coordination and coma in severe cases) and gastrointestinal symptoms (nausea, vomiting, abdominal pain and diarrhea). Respiratory problems and eye irritation can occur	General supportive care, bronchodilators, fluid replacement Gastrointestinal decontamination with activated charcoal for patients with recent ingestion of toxin. Administration of sedatives and pain mitigation
TTX poisoning	Pufferfish, goby fish, gastropods, crabs and bivalves	Symptoms occur within 10–45 min, sometimes delayed to 3–6 h but human data on recovery are very variable	Numbness, paraesthesia, incoordination, severe respiratory failure, hypotension, cardiac dysrhythmias and death in fatal cases	There is no specific antidote. Treatment is supportive, with removal of unabsorbed toxin. Treatment options included cysteine, cholinesterase inhibitors, naloxone and steroids
Diarrheic shellfish poisoning (DSP)	Mussels, oysters, scallops, clams, cockles	Symptoms occur from 30 min to 5 h and continue for about 2–3 days	Diarrhea, gastrointestinal distress, nausea, vomiting, and abdominal pain	Replacement of electrolyte and fluid loss
Azaspiracid poisoning (AZP)	Mussels, oysters, scallops, clams, cockles	Onset: 3 h Duration: 15 h	Diarrhea, nausea, vomiting, and abdominal cramps	Replacement of electrolyte and fluid loss
Palytoxin poisoning	Fish, sea urchin, molluscs, crabs, octopus	Onset: Minutes to several hours	Bitter taste, oral and limb numbness, dizziness, myalgia, rabdomyolysis	Supportive care

**Table 4:** Current regulatory levels of marine toxins in seafood.

Seafood Poisoning	Representative Toxin/s	Regulated Levels in Seafood
Paralytic shellfish poisoning (PSP)	Saxitoxin (STX (2HCl))	0.8 mg STX eq/kg
Amnesic shellfish poisoning (ASP)	Domoic acid (DA) and isoDA	20 mg DA eq/kg
Ciguatera fish poisoning (CFP)	Pacific-ciguatoxin-1 (P-CTX-1) Caribbean-ciguatoxin-1 (C-CTX-1)	Absence (EU) 10 ng P-CTX-1 eq/kg (USA) 100 ng C-CTX-1/kg (USA) 0.025 MU/g (Japan, Mexico)
Neurotoxin shellfish poisoning (NSP)	_	20 MU/100 g
TTX poisoning	_	Absence (EU)
Diarrheic shellfish poisoning (DSP)	Okadaic acid (OA)	0.16 mg OA eq/kg
Azaspiracid poisoning (AZP)	Azaspiracid	0.16 mg AZA eq/kg
Palytoxin poisoning	_	_
